# Association Between Diabetes Mellitus and CT-Measured Optic Nerve Sheath Diameter: A Retrospective Observational Study

**DOI:** 10.7759/cureus.116121

**Published:** 2026-09-11

**Authors:** Ajay Chand P., Bibin Joseph, Eldhos Jacob, Arshiya Begum, Muhammed Ajmal T.

**Affiliations:** 1 Medical Imaging Technology, Father Muller College of Allied Health Sciences, Mangaluru, IND; 2 Medical Imaging Technology, Faculty of Physiotherapy and Allied Health Sciences, M.S. Ramaiah University of Applied Sciences, Bengaluru, IND; 3 Respiratory Medicine, Father Muller Medical College Hospital, Mangaluru, IND; 4 Radio Diagnosis, Unity Hospital, Mangaluru, IND

**Keywords:** computed tomography, diabetes mellitus, medical imaging, neuroimaging, ocular imaging, optic nerve, optic nerve sheath diameter, retrospective study

## Abstract

Background: Diabetes mellitus is associated with microvascular, neurological, and ocular complications. The optic nerve sheath contains a cerebrospinal-fluid-containing subarachnoid space and can be assessed quantitatively on imaging. However, evidence regarding differences in CT-measured optic nerve sheath diameter (ONSD) between diabetic and non-diabetic patients remains limited.

Objective: To evaluate the association between diabetes mellitus and ONSD measured by CT and determine whether significant differences exist between diabetic and non-diabetic individuals.

Methods: This retrospective observational study included 104 patients aged 18-85 years who underwent non-contrast brain CT between June 2022 and June 2023. The sample comprised 52 diabetic and 52 non-diabetic patients. ONSD was measured bilaterally at 3 mm and 8 mm posterior to the globe using electronic calipers on the Syngo.via workstation. Patients with intracranial, orbital, or optic nerve conditions likely to affect ONSD were excluded. Group differences were evaluated using independent-samples t-tests. Multivariable linear regression models were additionally used to assess the association between diabetes status and ONSD after adjustment for age and sex.

Results: Mean age was 56.50 ± 13.08 years in the diabetic group and 45.94 ± 16.57 years in the non-diabetic group. Mean ONSD was greater in diabetic patients at all four sites: right 3 mm, 6.09 ± 0.59 versus 4.39 ± 0.53 mm; left 3 mm, 5.93 ± 0.65 versus 4.52 ± 0.62 mm; right 8 mm, 4.86 ± 0.57 versus 3.73 ± 0.48 mm; and left 8 mm, 4.89 ± 0.59 versus 3.78 ± 0.49 mm. All unadjusted comparisons were statistically significant (t = 15.46, 11.32, 10.93, and 10.44, respectively; all p < 0.001). After adjustment for age and sex, diabetes remained significantly associated with greater ONSD at all four sites.

Conclusion: Diabetic patients had larger CT-measured ONSD than non-diabetic patients at both 3 mm and 8 mm posterior to the globe. The association remained significant after adjustment for age and sex. These findings support an association between diabetes mellitus and increased ONSD measurements but do not establish causality or a diagnostic cutoff. Prospective studies with standardized measurements and detailed clinical characterization are warranted.

## Introduction

Diabetes mellitus is a chronic metabolic disorder characterized by persistent hyperglycemia resulting from impaired insulin secretion, impaired insulin action, or both. It is associated with systemic complications involving the cardiovascular, renal, neurological, and ocular systems. Chronic hyperglycemia can contribute to microvascular injury, endothelial dysfunction, oxidative stress, and inflammatory changes, which may affect neural and vascular tissues [1].

The optic nerve consists of axons of retinal ganglion cells that transmit visual information from the retina to the brain. The optic nerve is surrounded by meningeal coverings, including a cerebrospinal-fluid-containing subarachnoid space. Because of this anatomical relationship, the optic nerve sheath can be influenced by changes in intracranial pressure and surrounding cerebrospinal fluid dynamics [2,3]. Optic nerve sheath diameter (ONSD) can therefore be assessed as an imaging parameter in several neurological and neuro-ophthalmic conditions.

Diabetes-related metabolic and microvascular abnormalities may potentially influence the optic nerve and its surrounding structures. Chronic hyperglycemia has also been associated with microvascular damage, endothelial dysfunction, oxidative stress, and inflammatory changes, while diabetic ocular complications, including diabetic retinopathy and diabetic macular edema, may affect ocular and visual function [1,4]. Previous studies have investigated ONSD in the setting of diabetes, particularly during acute hyperglycemic states and diabetic ketoacidosis (DKA). Studies in patients with DKA have explored ONSD as a potential marker of cerebral edema and changes in intracranial pressure, particularly in pediatric populations [5,6]. In adults with acute hyperglycemic complications, Erol et al. evaluated ultrasonographic ONSD and reported that pretreatment ONSD did not differ significantly from that of healthy controls, although ONSD changed following treatment [7]. These findings suggest that the relationship between diabetes, hyperglycemia, and ONSD may vary according to the clinical context, severity of metabolic disturbance, imaging modality, and measurement conditions. However, these previous investigations have predominantly used ultrasonography and have focused on acute hyperglycemic states or DKA. The relationship between diabetes mellitus in a broader clinical population and CT-measured ONSD therefore remains insufficiently characterized.

CT is widely available and provides detailed visualization of the orbital and intracranial structures. ONSD can be measured on axial CT images at standardized distances posterior to the globe. Vaiman et al. evaluated 300 adults using CT and measured ONSD at 3 mm and 8 mm posterior to the globe, demonstrating measurable variation according to the anatomical location [8]. These standardized measurement sites have subsequently been used in CT-based assessment of ONSD [8,9].

ONSD has been investigated as a non-invasive imaging marker of raised intracranial pressure because the optic nerve sheath is anatomically continuous with the intracranial subarachnoid space [10]. Changes in intracranial pressure may therefore influence the diameter of the optic nerve sheath. However, ONSD measurements may vary according to the imaging modality, anatomical measurement site, measurement technique, and characteristics of the study population [8,9]. These variations highlight the importance of standardized measurement protocols when interpreting ONSD values across different studies.

Although ONSD has been studied extensively in relation to raised intracranial pressure and other neurological conditions, relatively few studies have specifically evaluated CT-measured ONSD in patients with diabetes mellitus. The potential influence of diabetes-related metabolic, microvascular, and neural changes on ONSD therefore remains insufficiently understood. Determining whether ONSD differs between diabetic and non-diabetic patients may provide additional information regarding possible structural changes involving the optic nerve sheath and may help generate hypotheses for future studies.

Therefore, the primary objective of this exploratory observational study was to compare CT-measured ONSD between diabetic and non-diabetic patients at 3 mm and 8 mm posterior to the globe. The secondary objective was to determine whether the observed association between diabetes mellitus and ONSD persisted after adjustment for age and sex. This hypothesis-generating study was not designed to establish causality or determine the clinical significance of increased ONSD.

## Materials and methods

This retrospective observational study was conducted in the Department of Radio-diagnosis and Imaging at A.J. Institute of Medical Sciences & Research Centre, Mangaluru, India. The study was based on previously available CT records and clinical information collected during the study period from June 2022 to June 2023.

Patients aged 18-85 years who underwent non-contrast CT brain examination were considered for inclusion, irrespective of sex. Eligible participants were retrospectively identified from available non-contrast CT brain examinations performed for various routine clinical indications and recorded in the hospital Picture Archiving and Communication System (PACS) during the study period. The corresponding clinical records were reviewed to determine diabetes status and assess the predefined inclusion and exclusion criteria. Patients with adequate axial CT images suitable for bilateral ONSD measurement and sufficient clinical information were included in the final analysis. The final study sample comprised 104 patients, including 52 patients with diabetes mellitus and 52 non-diabetic patients. The groups were not individually matched, and participants were not randomly selected.

Diabetes mellitus status was determined from the documented clinical diagnosis in the hospital medical records. Patients with a documented diagnosis of diabetes mellitus were classified as diabetic, whereas patients without a documented diagnosis of diabetes mellitus were classified as non-diabetic. Diabetes classification was based on the recorded clinical diagnosis and was not determined using a study-specific blood glucose or HbA1c threshold.

Patients were included if adequate axial CT images were available for bilateral ONSD measurement. Patients with intracranial neoplasms, cranial deformity or fracture, previous invasive cranial procedures, known optic nerve impairment, ocular or orbital injury, diabetic retinopathy, optic neuropathy, or other documented ocular conditions likely to affect ONSD measurements were excluded. The presence or absence of these conditions was determined from the available clinical and ophthalmological information documented in the medical records.

CT examinations were performed using a Siemens SOMATOM go.Top dual-source 128-slice CT scanner (Siemens Healthineers, Germany). Images were reviewed and ONSD measurements were performed using the syngo.via workstation (Siemens Healthineers, Germany). The specific software version used for syngo.via was not available in the retrospective study records and therefore could not be retrospectively verified.

ONSD was measured bilaterally on axial CT images at two predefined locations, 3 mm and 8 mm posterior to the globe, using electronic calipers. The posterior aspect of the globe was used as the anatomical reference point, and the specified distances were determined posteriorly from this reference point before measuring the ONSD. Measurements were obtained using the same anatomical reference and measurement approach for all participants. The two measurement locations were selected based on their use in previous CT-based ONSD studies and allowed assessment of the optic nerve sheath at different distances posterior to the globe. The ONSD was measured across the visible optic nerve sheath at each specified measurement location. The original retrospective records did not contain standardized documentation of the exact CT window width and window level, caliper-edge convention, or precise measurement-axis orientation used specifically for ONSD measurement; therefore, these technical parameters could not be retrospectively verified. Representative measurements are shown in Figure 1.

**Figure 1:**
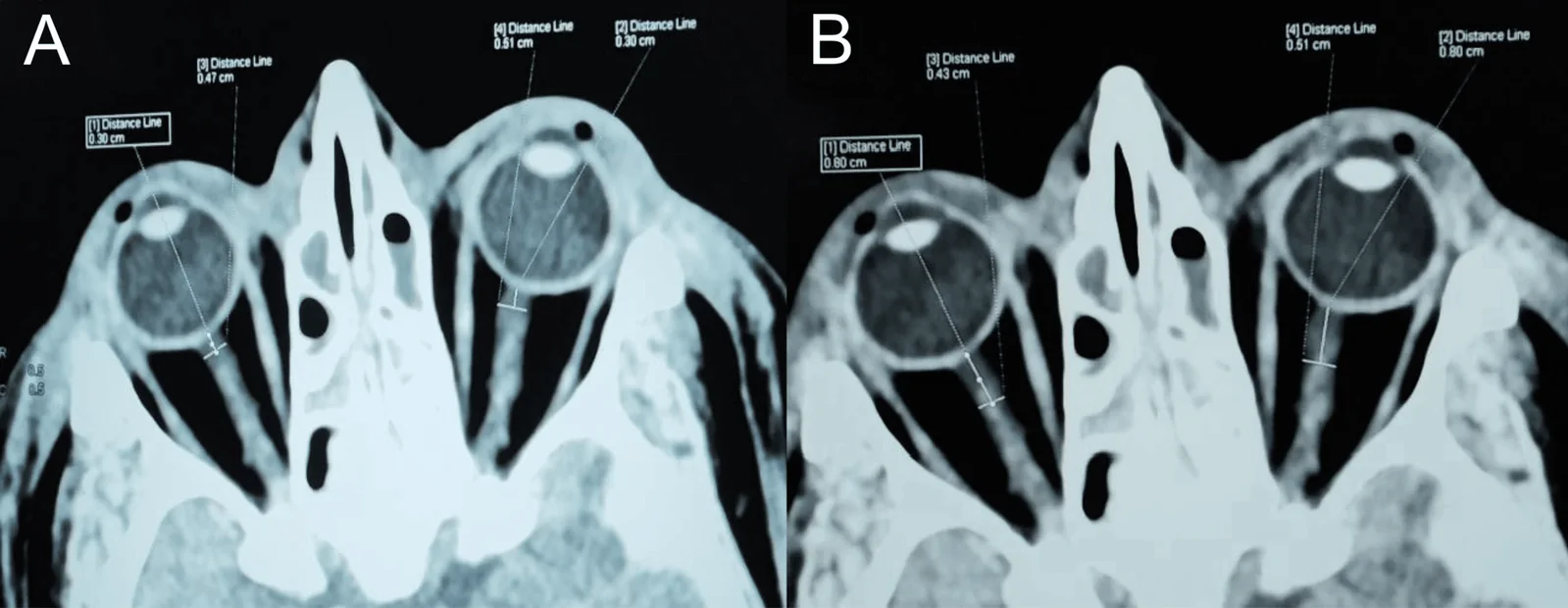
Axial CT measurement of ONSD (A) Bilateral ONSD measurement at 3 mm posterior to the globe; (B) Bilateral ONSD measurement at 8 mm posterior to the globe Measurements were obtained using electronic calipers with the posterior margin of the globe used as the anatomical reference point. ONSD: Optic nerve sheath diameter

Data were retrieved from the hospital PACS and corresponding clinical records. During retrospective review of the available CT examinations, the images were assessed for major intracranial or orbital abnormalities that could potentially affect ONSD measurements, in addition to applying the predefined exclusion criteria. A formal or quantitative assessment of imaging markers of intracranial hypertension was not performed. Demographic information, diabetes status, available random blood glucose measurements, and bilateral ONSD measurements were recorded in a structured data sheet. Random blood glucose values were extracted when available in the clinical records. Because random blood glucose measurements were not routinely available for all participants, this variable was treated as an incompletely observed descriptive clinical characteristic and was not used as an eligibility criterion or covariate in the primary regression analyses.

The study was reviewed and approved by the Institutional Ethics Committee, A.J. Institute of Medical Sciences & Research Centre, Mangaluru, under approval number AJEC/REV/153/2022 dated August 17, 2022.

Continuous variables were summarized as mean and SD. Normality was assessed using the Kolmogorov-Smirnov and Shapiro-Wilk tests. Differences in ONSD between diabetic and non-diabetic groups were assessed using independent-samples t-tests. Four related ONSD outcomes were evaluated, comprising right and left ONSD measurements at 3 mm and 8 mm posterior to the globe. These measurement sites were predefined as part of the study objectives and were analyzed separately. No formal adjustment for multiple comparisons was prespecified in the original analysis plan. Mean differences with 95% confidence intervals (CIs) and Cohen’s d effect sizes were reported. Because the diabetic and non-diabetic groups differed substantially in age, with the diabetic group being approximately 10 years older, multivariable linear regression models were performed separately for right and left ONSD measurements at 3 mm and 8 mm, with diabetes status, age, and sex included as explanatory variables. The regression models were used to estimate the association between diabetes status and ONSD after adjustment for age and sex. Detailed model-fit statistics and regression diagnostic measures from the original retrospective analysis were not retained and therefore could not be retrospectively verified. The adjusted regression coefficient for diabetes status represented the estimated difference in ONSD between diabetic and non-diabetic patients after adjustment for age and sex. However, other potentially relevant factors, including hypertension, obesity or body mass index, renal disease, systemic vascular disease, CT indication, and other neurological or ophthalmological conditions, were not systematically available or included in the regression models and may therefore represent sources of residual confounding. A two-sided p-value of <0.05 was considered statistically significant. Statistical analyses were performed using IBM SPSS Statistics for Windows, Version 20.0 (IBM Corp., USA). 

## Results

A total of 104 patients were included in the analysis, comprising 52 patients with diabetes mellitus and 52 non-diabetic patients. The diabetic group was older than the non-diabetic group, with mean ages of 56.50 ± 13.08 years and 45.94 ± 16.57 years, respectively, while the sex distribution was similar between groups. Baseline demographic and available blood glucose characteristics are presented in Table 1. The distributions of the ONSD measurements were assessed using the Kolmogorov-Smirnov and Shapiro-Wilk tests. The ONSD measurements were considered sufficiently normally distributed to satisfy the assumptions for the subsequent parametric analyses.

**Table 1:** Baseline characteristics of diabetic and non-diabetic participants *Random blood sugar measurements were available for 35 diabetic and 48 non-diabetic participants.

Characteristic	Diabetic (n=52)	Non-diabetic (n=52)
Age, mean ± SD (years)	56.50 ± 13.08	45.94 ± 16.57
Male, n (%)	43 (82.7)	41 (78.8)
Female, n (%)	9 (17.3)	11 (21.2)
Random blood sugar, mean ± SD (mg/dL)*	276.83 ± 77.18	127.04 ± 30.44

Comparison of ONSD at 3 mm

At 3 mm posterior to the globe, the mean right ONSD was 6.09 ± 0.59 mm in diabetic patients compared with 4.39 ± 0.53 mm in non-diabetic patients. The mean difference was 1.70 mm (95% CI, 1.49-1.92; t = 15.46, p < 0.001)

The mean left ONSD was 5.93 ± 0.65 mm in diabetic patients compared with 4.52 ± 0.62 mm in non-diabetic patients. The mean difference was 1.41 mm (95% CI, 1.17-1.66; t = 11.32, p < 0.001).

Comparison of ONSD at 8 mm

At 8 mm posterior to the globe, the mean right ONSD was 4.86 ± 0.57 mm in diabetic patients compared with 3.73 ± 0.48 mm in non-diabetic patients (t = 10.93, p < 0.001).

The mean left ONSD was 4.89 ± 0.59 mm in diabetic patients compared with 3.78 ± 0.49 mm in non-diabetic patients (t = 10.44, p < 0.001). These findings are illustrated in Figure 2. The comparisons of ONSD between diabetic and non-diabetic patients are summarized in Table 2.

**Figure 2:**
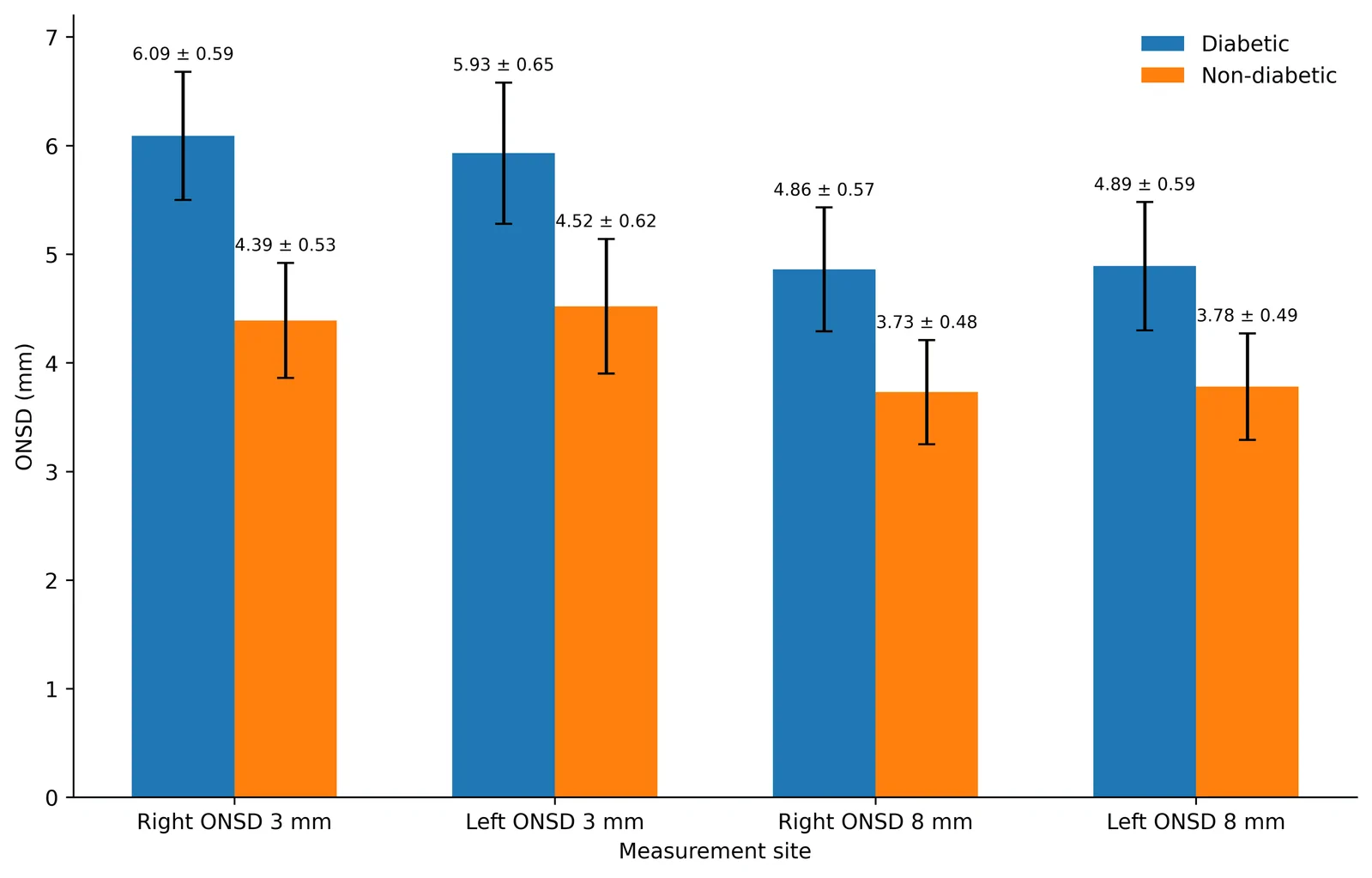
Mean CT-measured ONSD in diabetic and non-diabetic patients Mean  ONSD measured at 3 mm and 8 mm posterior to the globe on axial CT images in diabetic and non-diabetic patients. Bars represent mean ONSD values, and error bars represent SD. Between-group comparisons were performed using independent-samples t-tests; statistical results are presented in Table 2. ONSD: Optic nerve sheath diameter

**Table 2:** Comparison of ONSD between diabetic and non-diabetic patients ONSD was measured bilaterally at 3 mm and 8 mm posterior to the globe on axial CT images. Mean differences represent the diabetic group minus the non-diabetic group. Statistical significance was assessed using independent-samples t-tests. Cohen’s d represents the standardized mean difference between groups. ONSD: Optic nerve sheath diameter; CI: Confidence interval

ONSD measurement	Diabetic, mean ± SD	Non-diabetic, mean ± SD	Mean difference (mm)	95% CI	Cohen’s d	t - statistics	p-value
Right ONSD at 3 mm	6.09 ± 0.59	4.39 ± 0.53	1.7	1.49–1.92	3.09	15.46	<0.001
Left ONSD at 3 mm	5.93 ± 0.65	4.52 ± 0.62	1.41	1.17–1.66	2.25	11.32	<0.001
Right ONSD at 8 mm	4.86 ± 0.57	3.73 ± 0.48	1.12	0.92–1.33	2.19	10.93	<0.001
Left ONSD at 8 mm	4.89 ± 0.59	3.78 ± 0.49	1.11	0.90–1.32	2.05	10.44	<0.001

Age- and sex-adjusted analysis

Because the diabetic group was older than the non-diabetic group, multivariable linear regression models were performed with diabetes status, age, and sex as explanatory variables. After adjustment for age and sex, diabetes remained significantly associated with greater ONSD at all four measurement locations.

For right ONSD at 3 mm, the adjusted diabetes coefficient was 1.74 mm (95% CI, 1.51-1.97; t = 14.99, p < 0.001). For left ONSD at 3 mm, the adjusted coefficient was 1.43 mm (95% CI, 1.16-1.69; t = 10.81, p < 0.001). At 8 mm, the adjusted coefficients were 1.06 mm (95% CI, 0.84-1.28; t = 9.46, p < 0.001) for the right side and 1.09 mm (95% CI, 0.86-1.32; t = 9.70, p < 0.001) for the left side. These findings are illustrated in Figure 3. The age- and sex-adjusted associations between diabetes mellitus and ONSD are presented in Table 3. 

**Figure 3:**
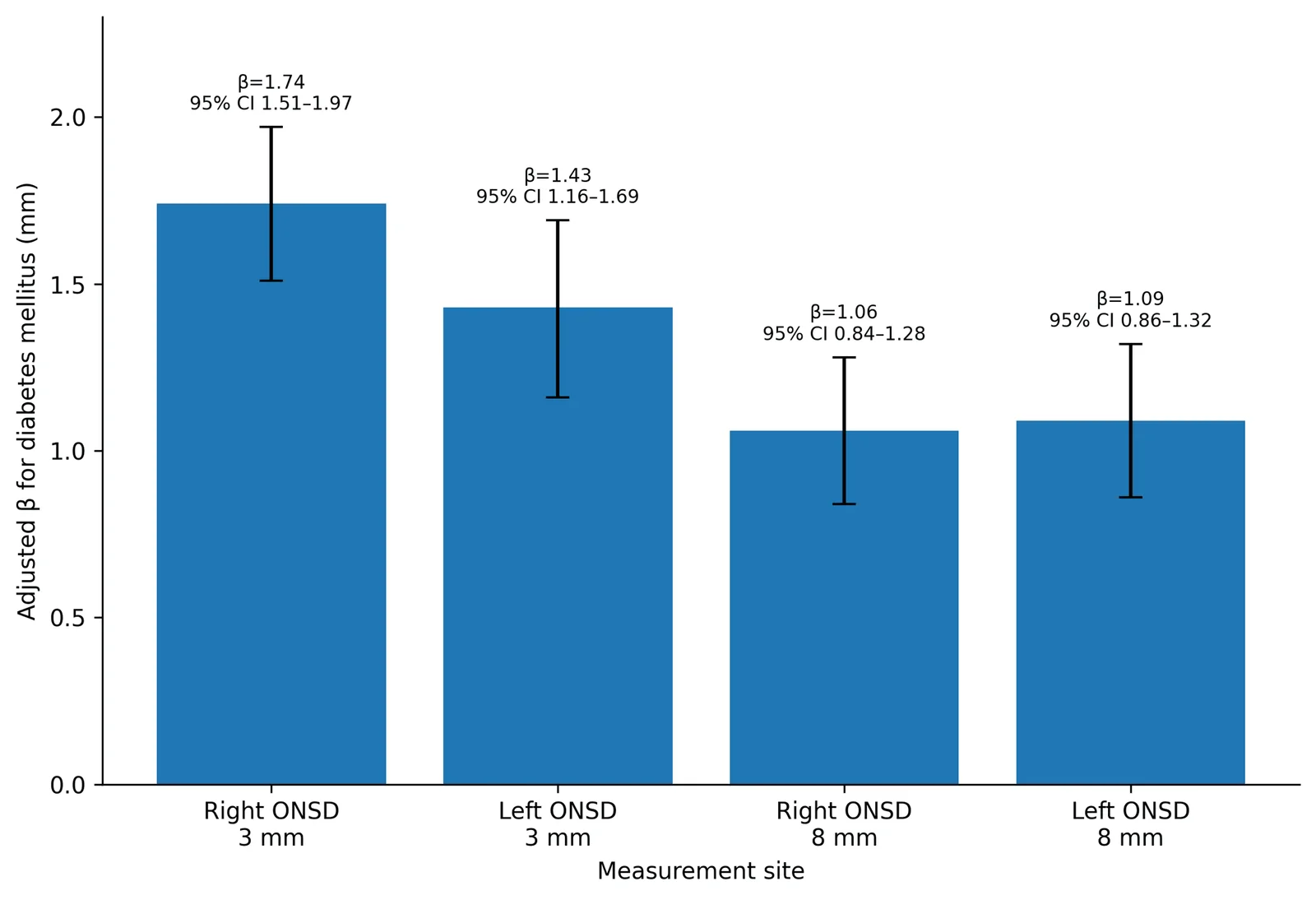
Age- and sex-adjusted association between diabetes mellitus and ONSD Adjusted regression coefficients (β) for the association between diabetes mellitus and ONSD at 3 mm and 8 mm posterior to the globe after adjustment for age and sex. Error bars represent 95% CIs. ONSD: Optic nerve sheath diameter; CI: Confidence interval

**Table 3:** Multivariable linear regression analysis of the association between diabetes mellitus and ONSD Multivariable linear regression models were adjusted for age and sex. The adjusted β represents the estimated difference in ONSD associated with diabetes mellitus after adjustment for age and sex. The t-statistic and two-sided p-value are reported for the diabetes mellitus coefficient. ONSD: Optic nerve sheath diameter; β: Regression coefficient; CI: confidence interval

ONSD measurement	Adjusted β (mm)	95% CI	t-statistic*	p-value
Right ONSD at 3 mm	1.74	1.51–1.97	14.99	<0.001
Left ONSD at 3 mm	1.43	1.16–1.69	10.81	<0.001
Right ONSD at 8 mm	1.06	0.84–1.28	9.46	<0.001
Left ONSD at 8 mm	1.09	0.86–1.32	9.7	<0.001

Blood glucose measurements

Random blood sugar measurements were available for 48 non-diabetic and 35 diabetic patients. The mean random blood sugar level was 127.04 ± 30.44 mg/dL in the non-diabetic group and 276.83 ± 77.18 mg/dL in the diabetic group. These values are presented descriptively because diabetes status was established from the clinical records and random blood glucose measurements were not available for all participants.

## Discussion

The present study found that ONSD measured on axial CT images was significantly greater in patients with diabetes mellitus than in non-diabetic patients at both 3 mm and 8 mm posterior to the globe, bilaterally. The association between diabetes mellitus and increased ONSD remained significant after adjustment for age and sex. These findings suggest that diabetes mellitus is associated with larger CT-measured ONSD. However, the findings should not be interpreted as evidence of raised intracranial pressure or as establishing a causal relationship.

The magnitude of the difference was greatest at the 3-mm measurement site. The mean right ONSD was 6.09 ± 0.59 mm in diabetic patients compared with 4.39 ± 0.53 mm in non-diabetic patients, while the corresponding left-sided measurements were 5.93 ± 0.65 mm and 4.52 ± 0.62 mm, respectively. At 8 mm posterior to the globe, the mean right and left ONSD measurements were also greater in diabetic patients. The observation of higher ONSD values across both eyes and both measurement locations indicates a consistent pattern in the study sample; however, this consistency should not be interpreted as evidence of measurement robustness because formal measurement reliability was not assessed and potentially relevant clinical confounders were unavailable. An important feature of this study was the adjustment for age and sex. The diabetic group was older than the non-diabetic group, making age an important potential confounder. After adjustment for age and sex, diabetes remained associated with larger ONSD at all four measurement locations. The adjusted differences were approximately 1.74 mm for right ONSD at 3 mm, 1.43 mm for left ONSD at 3 mm, 1.06 mm for right ONSD at 8 mm, and 1.09 mm for left ONSD at 8 mm. These findings indicate that the association between diabetes mellitus and ONSD persisted after adjustment for age and sex; however, residual confounding from other potentially relevant clinical factors cannot be excluded.

Previous literature specifically examining diabetes and ONSD has largely focused on acute hyperglycemic complications, particularly DKA and cerebral edema. Studies in children with DKA have investigated ONSD as a potential non-invasive marker of cerebral edema, with ONSD measurements changing during metabolic treatment [5,6]. In adults with acute hyperglycemic complications, Erol et al. reported no significant difference in pretreatment ultrasonographic ONSD between diabetic patients and healthy controls, although ONSD increased after treatment [7]. These findings differ from the present study, in which patients with diabetes mellitus demonstrated larger CT-measured ONSD than non-diabetic participants. Differences in patient population, clinical setting, severity of hyperglycemia, imaging modality, measurement technique, and timing of ONSD assessment may contribute to these differences. Importantly, the present study evaluated patients with diabetes mellitus identified from routine clinical records rather than restricting the population to acute diabetic emergencies. Therefore, these findings add to the existing literature by examining the association between diabetes mellitus and CT-measured ONSD outside the specific context of DKA or acute hyperglycemic complications.

Diabetes mellitus is associated with chronic microvascular injury, endothelial dysfunction, oxidative stress, and inflammatory changes, which may affect neural and vascular tissues [1,4]. The optic nerve and its surrounding structures may potentially be affected by such chronic systemic changes. However, the present study did not directly evaluate retinal microvascular changes, optic nerve perfusion, intracranial pressure, cerebrospinal fluid dynamics, or histopathological changes. Therefore, these mechanisms should be regarded as possible explanations rather than established causes of the increased ONSD observed in this study.

ONSD measurement has been widely investigated as an imaging marker in the assessment of raised intracranial pressure. Previous CT-based work has established standardized ONSD measurement sites and demonstrated the feasibility of CT-based ONSD assessment [8,9]. Vaiman et al. reported normative CT measurements of ONSD in healthy adults [8], while Jalayondeja et al. evaluated the relationship between CT-measured ONSD and intracranial pressure [9].

The present findings should not be interpreted as evidence that increased ONSD in patients with diabetes necessarily indicates raised intracranial pressure. ONSD can be influenced by several physiological and pathological factors, and the present study did not directly measure intracranial pressure. Previous studies using ultrasound have demonstrated the utility of ONSD measurement in identifying raised intracranial pressure [10]. However, those findings cannot be directly extrapolated to the diabetic population studied here. Rather, the findings of the present study indicate an association between diabetes mellitus and larger CT-measured ONSD.

MRI-based studies have also established standardized approaches for assessing optic nerve and optic nerve sheath dimensions [11-13]. These findings further emphasize the importance of standardized imaging techniques when evaluating ONSD across different modalities. Recent studies have further supported the clinical utility of ONSD measurement, particularly using ultrasonography, while emphasizing the importance of standardized measurement techniques and recognition of interobserver and methodological variability [14].

The available blood glucose findings provide additional clinical context. Blood glucose levels were substantially higher among diabetic participants than among non-diabetic participants. However, biochemical measurements were not available for all participants, and therefore these data should be interpreted as supportive descriptive information rather than as evidence of a dose-response relationship between glycemic status and ONSD. Further studies with complete and standardized measurements of glycated hemoglobin and other metabolic variables would be useful to investigate whether the magnitude or duration of hyperglycemia is associated with ONSD.

This study has several strengths. The diabetic and non-diabetic groups contained equal numbers of participants, and ONSD was assessed bilaterally at two standardized anatomical locations. The use of routinely acquired CT examinations allowed retrospective quantitative assessment of the orbital structures; however, CT-based ONSD measurements may be influenced by spatial resolution, partial-volume effects, and observer-dependent caliper placement. The persistence of the observed association after adjustment for age and sex provides additional support for the primary finding, though residual confounding cannot be excluded.

Several limitations should be considered. First, the retrospective single-center design limits the ability to establish causality and may introduce selection bias. The clinical indications for CT examination may have differed between participants and could represent a potential source of residual confounding. Because the study was retrospective, CT indications were not systematically incorporated into the statistical models. The exclusion of patients with documented diabetic retinopathy and optic neuropathy may limit the generalizability of the findings to the broader population of patients with diabetes mellitus, particularly those with established diabetic ocular complications.

Second, the diabetic group was approximately 10 years older than the non-diabetic group, representing a substantial baseline imbalance and an important potential source of confounding. Although age was included as a continuous covariate in the multivariable regression models, a formally documented assessment of the linearity of the relationship between age and ONSD was not available from the retrospective analysis. Therefore, residual confounding by age cannot be completely excluded. Complete regression diagnostic information, including model-fit statistics and formal assessment of all relevant linear regression assumptions, was not retained from the original analysis. Consequently, the adequacy of the regression model assumptions cannot be fully verified retrospectively. Adjustment for potential confounding was limited to age and sex. Other factors that may be associated with diabetes mellitus and ONSD, including hypertension, obesity, renal disease, clinical indication for CT examination, and other systemic conditions, were not systematically available or incorporated into the regression models. Therefore, residual confounding from these factors cannot be excluded.

Third, information regarding the duration of diabetes, glycemic control, diabetic complications, and antidiabetic treatment was limited or unavailable. Additionally, patients with diabetic retinopathy were excluded to minimize potential ocular factors that could influence ONSD measurements. This exclusion may limit the generalizability of the findings to the broader diabetic population, particularly patients with established diabetic microvascular ocular complications. Random blood glucose measurements were incomplete because testing was not uniformly documented in the retrospective clinical records. Values were available for 35 of 52 diabetic participants and 48 of 52 non-diabetic participants, indicating unequal availability between groups. Because the reasons for testing and missingness were not systematically documented, systematic differences in glucose-data availability cannot be excluded. Glucose was therefore treated only as descriptive clinical information and was not used to define diabetes status or as a covariate in the primary analyses.

Fourth, interobserver and intraobserver measurement reliability was not assessed. Additionally, certain technical parameters of the original CT-based measurement, including the exact window settings, caliper-edge convention, and measurement-axis orientation, could not be retrospectively verified. These factors may contribute to measurement variability.

Fifth, because of the retrospective nature of the study, a complete screening log documenting the total number of CT examinations reviewed and the number of exclusions at each stage was not available.

Sixth, four related ONSD outcomes were evaluated without a prespecified adjustment for multiple comparisons; therefore, the possibility of an increased type I error cannot be excluded, and the findings should be interpreted cautiously.

Seventh, although the CT examinations were reviewed for major intracranial and orbital abnormalities, imaging findings suggestive of intracranial hypertension were not systematically assessed or quantified. Therefore, unrecognized intracranial pressure-related factors cannot be completely excluded. Finally, intracranial pressure was not directly measured, and therefore the increased ONSD measurements cannot be interpreted as evidence of intracranial hypertension.

Future prospective, multicenter studies should include standardized ONSD measurement protocols, assessment by multiple observers, evaluation of interobserver and intraobserver reliability, and comprehensive characterization of diabetes duration and glycemic control. Correlation with ophthalmological findings, retinal vascular changes, and direct or validated surrogate measures of intracranial pressure may also help clarify the clinical significance of increased ONSD in patients with diabetes. Such studies should also consider potential confounders including hypertension, body mass index, renal disease, CT indication, and other systemic conditions, and should prospectively document imaging acquisition and measurement parameters.

## Conclusions

This retrospective study found that CT-measured ONSD was significantly greater in patients with diabetes mellitus than in non-diabetic patients at both 3 mm and 8 mm posterior to the globe, bilaterally. The association between diabetes mellitus and increased ONSD remained significant after adjustment for age and sex. These findings suggest that diabetes mellitus is associated with larger CT-measured ONSD. However, the findings should not be interpreted as evidence of raised intracranial pressure or as establishing a causal relationship. Further prospective, multicenter studies incorporating standardized ONSD measurement protocols, assessment of measurement reliability, detailed characterization of diabetes duration and glycemic control, and relevant clinical correlates are warranted to determine the clinical significance of this association.
